# The experiences, unmet needs and outcomes of parents of severely injured children: a longitudinal mixed methods study protocol

**DOI:** 10.1186/s12887-016-0693-8

**Published:** 2016-09-06

**Authors:** Kim Foster, Kate Curtis, Rebecca Mitchell, Connie Van, Alexandra Young

**Affiliations:** 1 Australian Catholic University, and Northwestern Mental Health, Melbourne Health, Level 1 North, City Campus, The Royal Melbourne Hospital, Grattan Street, Parkville, VIC 3050 Australia; 2 Sydney Nursing School, The University of Sydney, 88 Mallett St, Camperdown, NSW 2006 Australia; 3 Trauma Service, St George Hospital, Gray St, Kogarah, NSW 2217 Australia; 4 Australian Institute of Health Innovation, Macquarie University, Level 6, 75 Talavera Road, Sydney, NSW 2109 Australia

**Keywords:** Parents, Children, Family, Trauma, Resilience, Psychological distress, Injury, Mixed Methods, Longitudinal

## Abstract

**Background:**

Being the parent of a severely injured child involves many stressors throughout the trauma journey. Internationally, little is known about the experiences or levels of emotional distress, parenting stress, quality of life, and resilience for parents of injured children. The aim of this study is to investigate the experiences, unmet needs and outcomes of parents of physically injured children 0–12 years over the 2 year period following injury.

**Methods/design:**

This is a prospective longitudinal study using an embedded mixed methods design. This design has a primary qualitative strand which incorporates supplementary quantitative data on child quality of life, and parental quality of life, parenting stress, emotional distress, and resilience at four time points; the acute hospitalisation phase, and at 6, 12 and 24 months following injury. The primary sample are parents of injured children 0–12 years hospitalised in the Australian states of New South Wales, Queensland, Victoria and South Australia. Primary data sources are child and parent demographic data; survey data; and semi-structured interview data across a 24 month period.

**Discussion:**

This study aims to address the existing gap in knowledge on the experiences and unmet support needs of parents in the 2 years following child injury to provide guidance for care provision for these families. There is a lack of evidence-based recommendations for supporting parents and families of injured children and strengthening their capacity to address the challenges they face.

## Background

Being the parent of a physically injured child involves many stressors throughout the trauma journey, including witnessing the pain, fear and often shocking physical changes in their child, seeing other injured children in hospital, and being under constant pressure to make difficult and traumatic decisions [1–3]. In the first days after their child’s injury, parental anxiety can be elevated to near panic levels [4], and more than 60 % of parents of children hospitalised after serious injury can meet the criteria for Acute Stress Disorder [5].

After the initial injury crisis passes, parents need to come to terms with the longer term implications of their child’s injury and learn about their care needs. During this time, parents experience emotions ranging from sadness and loneliness to feelings of shock, grief, guilt and helplessness [6–8]. Around 20–40 % of parents are at risk of developing depression or anxiety after the injury [9]. Parents without the skills or support to manage these emotions are at clear risk of psychological distress. While it depends on the type and severity of the injury, research has identified that around 10–30 % of parents of critically injured children develop post-traumatic stress disorder (PTSD) after their child’s injury [6, 10, 11]. Research has also found that 20–40 % of parents are at risk of developing depression or anxiety after the injury [10].

The entire family can be severely impacted by a child’s injury [10] and parents’ relationships are also placed under strain [10, 11]. Children depend on their parents to take care of their physical, emotional, and practical needs. A parent’s ability to cope with the stress associated with the child’s injury can affect the quality of life of their children and all members of the family [12]. It has also been reported that serious injury in a child can have a negative impact on family dynamics [10] and can threaten the wellbeing of the entire family unit [13, 14]. Despite the common assumption that this stress can cause divorce, parents’ relationships can also be remarkably resilient when supported well, with reports that a child’s serious injury does not always lead to problems in marital adjustment and family functioning [15, 9, 16].

From an Australian perspective, little is known about the experiences or levels of emotional distress, parenting stress, quality of life, and resilience for parents of injured children. There is a subsequent lack of evidence-based recommendations or solutions for supporting parents and families of injured children and strengthening their capacity to address the challenges they face. A consensus meeting of European trauma experts on quality of life after multiple trauma [17] indicated the assessment of long-term outcomes should take place at 2 years post-injury, with interim assessments recommended during this time.

This study aims to investigate the experiences, unmet needs and outcomes of parents of severely injured children 0–12 years over the 2 year period following injury. More specifically the study aims to:

1. Explore parents’ experiences of parenting an injured child in the acute hospitalisation phase, and at 6, 12, and 24 months following injury;
2. Identify parents’ unmet needs and factors that contribute to, or impede, needs being met during the 24 months following injury; and
3. Measure child and parent quality of life, parental emotional distress, parenting stress, and resilience during the acute hospitalisation phase, and at 6, 12 and 24 months following injury.

## Methods/design

This is a prospective longitudinal study using an embedded mixed methods design [18]. This mixed methods design has a primary qualitative strand (QUAL) which incorporates supplementary quantitative (quan) data on child and parental quality of life (QoL) and parenting stress, emotional distress, and resilience, at each of the four study time points. This is a useful design for mixing different data sets where one form of data is embedded within the overall study design to supplement the primary form of data [18]. In this study therefore, the primary focus is the qualitative data on parent experiences.

An interpretive qualitative approach will guide the primary qualitative strand. This form of inquiry recognises the self-reflective nature of qualitative research and the key role of the researcher in interpreting the data and the meanings attributed to experience [19]. This inductive approach is appropriate when there is little known about a phenomenon, and where the intention is to build theory [20].

### Sample and setting

The study sites comprise five of the six major trauma services for children in Australia: Up to 40 parents of severely injured children 0–12 years hospitalised for their injury in New South Wales, South Australia, Queensland and Victoria will be purposively selected and form the longitudinal study sample.

A purposive sample is used in qualitative research as individuals and sites are selected because they can purposefully inform understandings of the study phenomenon [19]. The sample size of 40 is appropriate for a study with a primary qualitative focus and allows for variation of experience across the study sites [21]. As this is a mixed methods longitudinal study, the sample size also allows for statistical analysis of the outcome measures and for participant loss to follow up which may occur. Final sample size will be assessed on an ongoing basis to identify when sufficient saturation of data is achieved. This occurs when further data sampling will not significantly contribute to expanding understandings of the phenomenon of interest [21].

### Participant enrolment

Trauma coordinators at each hospital will screen and approach potential parent participants. The Trauma Coordinator is an experienced clinician, whose role is to coordinate inpatient care, collect trauma data and provide staff with inpatient education. They also oversee quality and performance improvement.

Potential parent participants will be identified by the Trauma Coordinator at each site during the clinical rounds and subsequently discussed with the clinical team in terms of suitability for the study. This will minimise the risk of coercion. Parent participants will be screened by the Trauma Coordinator according to the inclusion criteria, informed of the study and invited to participate. They will be provided with a participant information package including the participant information sheet, consent form, the suite of measures and return envelopes for each of the 4 time points of the study. Parents will be asked for their preferred mode of contact (eg email, phone call, text) for follow-up contact by the Study Coordinator.

The *inclusion criteria* for participants in the study are: parents must be over 18 years of age; able to speak, read and write English, and be parents of a recently injured and hospitalised child 0–12 years with an Injury Severity Score (ISS) greater than 15 or requiring admission to the Intensive Care Unit (ICU). The ISS is a globally used scoring system to quantify severity of injury [22]. All participants will provide written informed consent prior to participating in the study.

Potential participants will be excluded from the study (*exclusion criteria*) if they are: parents under 18 years of age; are non-English speaking, and the ISS for the child’s injury is less than 15. The researchers will not be contacting the child or any siblings.

### Ethical Considerations

Participants are not anticipated to experience adverse effects from direct participation in the study; however, it is possible they may experience emotional distress during the qualitative interviews. The interviewing investigators will be trained in appropriate interview techniques and in management of participant distress prior to interview commencement. In the information sheet that outlines their participation in the study, potential participants will be provided with contact details for emotional support services. They will also be advised to contact the principal investigator should they have any concerns during the course of the study or if they no longer wish to participate in the study. At any stage during any of the interviews, the participants will be free to stop and discontinue with any further questioning. No penalty will apply for withdrawal from the study.

There will not be a clearly defined threshold at which participants will be offered emotional support as interviews will not be used as a diagnostic tool. If a participant becomes distressed at any stage during the interviews, the following procedure will be followed:

1. Interview questioning will stop.
2. Emotional support will be provided by the investigator.
3. Participants will be asked if they wish to continue. If yes, the interview will continue. If not, the interview will cease and they will be asked if a loved one or other person is available to be with them for support, and referred to the support information provided in the participant information sheet. Participants will then be asked for permission for the investigator to come back and follow-up at another time.
4. Participants will be encouraged, if relevant, to see their GP for follow-up. If they are reluctant to contact their GP, and their distress is of concern, the investigator will ask the participant’s permission to contact their GP, or specialist, on their behalf. After ending the conversation, the participant’s GP will be contacted and informed of the incident, and any concerns the investigator may have of the participant’s emotional/mental status.

Ethics approval for the study has been sought and granted from the relevant University (HREC/13/SCHN/404), and the ethics committees at the respective hospital sites: (HREC/13/SCHN/404); (HREC/14/QRCH/149); (34089 A); and (HREC/14/WCHN/84).

### Quantitative data collection

Once parental consent is gained, child injury and outcome information will be obtained by the Trauma Coordinator from the hospital records: child age, gender, country of birth, type of injury, mechanism of injury, severity of injury, length of time since injury, operations, ICU length of stay and hospital length of stay. Consenting parents will be invited to complete a demographic information sheet and baseline measures and return them in a sealed envelope to the Trauma Coordinator.

Parent demographic information will be collected from parents: Parental age and gender, postcode, travel time to hospital, country of birth, marital status, number and age of other children, parental education, employment status and household income. Basic demographic information on potential participants who refuse to participate and the number of non-English speaking parents not asked to participate will also be gathered to enable examination of the study sample representativeness.

The following supplementary self-report standardised measures will be used during the acute hospitalisation phase, and at 6, 12 and 24 months: levels of child and parent quality of life, parental emotional distress, parenting stress, and resilience. These will take parents approximately 30–45 min to complete:

- Pediatric Inventory for Parents (PIP)

The PIP is a 42 item self-report measure, measuring parents’ experiences of stress or anxiety that are related to their children’s illness or injury. The 42 items are grouped into domain scales—communication, emotional functioning, medical care and role function. The internal consistency reliability for the PIP is Cronbach’s alpha 0.80–0.96 [23].

- Parent Quality of Life (SF 12v2)

The SF-12 is a 12 item self-report quality of life measure with physical and mental health items. It is well-validated and has Australian normative means [24].

- Depression, Anxiety & Stress (DASS 21)

The DASS-21 is a well-validated, 21 item self-report screening tool for depression, anxiety and stress within the past week, with Australian normative means [25].

- Connor Davidson resilience scale (CD-RISC)

The CD-RISC is a 25 item self-report measure of resilience in adults and has sound psychometric properties, and is capable of distinguishing between people with greater and lesser resilience. Prior testing shows good internal consistency and test-retest reliability with a Cronbach’s alpha of 0.89 indicating adequate consistency [26, 27].

- EQ-5D-Y

The EQ-5D-Y is a standardised measure for use as a health outcome measure of health conditions and populations. The EQ-5D-Y can be used as a parent proxy measure (as in this study) to explore how parents think their child would rate his/her own health. Parents answer as they believe the child would respond if he/she were able to fill in the measure his/herself. It consists of five dimensions: mobility, self-care, usual activities, pain or discomfort, and anxiety or depression, using ratings from 1 = ‘no problems’, 2 = ‘some problems’, to 3 = ‘extreme problems’. Parents also rate on a visual scale from 0 to 100 how they think their child would rate his/her health on the day [28].

### Qualitative data collection

The primary strand involves interviewing up to 40 parents of severely injured children 0–12 years to understand their perspectives on their child’s injury and the care they receive, and to determine their needs and what aspects of care they considered improved, or could improve, their experience within the first 2 years post injury. The primary aims of this strand will be to identify potential periods of vulnerability for parents and families, to track parents’ emotional wellbeing over time, and to ascertain the factors that support or impede their ability to manage their child’s injury.

Following completion of the outcome measures (supplementary quantitative strand) parent contact details will be provided to the Study Coordinator, who will liaise with the Trauma Coordinator regarding the appropriate timing for parent interviews (anticipated to be within the initial 3 weeks/post-injury). The Study Coordinator will contact parents and arrange to interview them (separately if more than one parent) at a mutually convenient time in a quiet and private setting in the hospital. In-depth face-to-face semi-structured interviews 45 min–1 h in length will be conducted. Follow-up telephone semi-structured interviews of up to 45 min will be conducted with the same parent at 6, 12 and 24 months post-injury.

Wherever possible, each parent will be encouraged to participate separately to provide more complete data on family needs. Interviews will explore parents’ experience following their child’s injury and, when relevant, will also allow direct comparisons of parents’ experiences and begin to delineate how best to meet the unique needs of each family member affected by the injury. Interview questions will elicit parents’ story, and explore their unmet support needs, such as information and practical needs. Interviews will also elicit parents’ suggestions as to what services they had (or would have) most valued at different time points. Interview questions are based on prior literature [29].

### Interviewer training

The principal and co-investigators are experienced researchers highly trained in quantitative and qualitative data collection and analysis. The Trauma Coordinator at each site are experienced clinicians, and will be trained in the study protocol and in interacting with and recruiting research participants prior to commencement of the study. They will be provided with ongoing support and regular contact from the investigators throughout the recruitment period.

The Study Coordinator is a trained researcher and will conduct the semi-structured qualitative interviews and analysis with the support of the investigators. Prior to conducting the interviews, the Study Coordinator will be carefully trained in research techniques specific to interacting with and recruiting research participants who may become distressed. The investigator will follow written guidelines delineating both the risks and safety for participants and the interviewer. For example, should a parent express distress, the Trauma Coordinator or the Study Coordinator will follow the protocol for the relevant support services and follow-up.

### Data confidentiality and data security

All study information will be maintained in the strictest confidence in accordance with the National Health and Medical Research Council (NHMRC) National Statement on Ethical Conduct in Research Involving Humans [30]. All results will be published in a form that will not allow individuals to be identified, that is, in de-identified aggregate form only; no individual results will be disclosed.

## Data analysis

### Quantitative data

Descriptive analysis will be conducted to examine quantitative measures data during the acute care phase. A repeated measures analysis will be used to compare parental responses over time between injury phases. As appropriate, comparison with population norms will also be conducted at each injury phase. If required, comparison between demographic characteristics of participants and non-participants (i.e. refusals) will be conducted using a t-test (or Mann-Whitney-Wilcoxon test, as appropriate) to compare continuous variables and a chi-square test of independence (or Fisher’s exact test, where relevant) for categorical variables.

### Qualitative data

The data collected from interviews will be transcribed verbatim and analysed using gold standard qualitative methodology, primarily that described by Miles & Huberman [31]. The software NVivo 10 (QSR International) will be used to facilitate the coding of participant interview responses. NVivo is a qualitative data analysis software program which also has capability to enable researchers to code and combine quantitative and qualitative data types, to matrix code, and to develop conceptual and theoretical modelling of data [32]. These are all relevant analytical functions for this mixed methods study. For the qualitative data, interview responses will be coded line by line, and codes will be grouped to form emerging categories and then developed into conceptual groups for the thematic organisation of responses [33].

### Interpretation

**Fig. 1:**
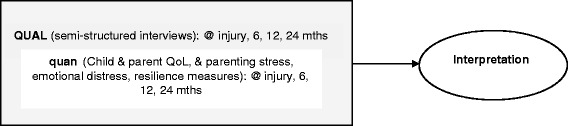
Embedded mixed methods design

As is appropriate in a mixed methods study, in the final interpretation phase of the study (See Fig. 1), qualitative and quantitative data will be combined and analysed in NVivo using matrix coding to cross-analyse participant response themes and clinical characteristics from the outcome measures on quality of life; emotional distress; parenting stress; and resilience.

## Discussion

The aim of this paper was to describe the protocol for the design of a study investigating the experiences, unmet needs, parenting stress, emotional distress, quality of life and resilience of Australian parents of injured children. This study will provide treating clinicians with information to use when providing care for injured children and their parents, and these findings can be used to influence the development of hospital policy and educational tools.

Identifying if barriers exist in the provision of care for injured children and their parents in the hospital settings will also help determine whether or not the existing care provided to families in the study settings is adequate. This information will be valuable in creating a better understanding of how best to address any existing barriers to care found as a result of talking to parents about their experiences of care in the hospital setting. The key principles of knowledge translation, for example involving the end users (parents and trauma clinicians) throughout, have been incorporated into the research design [34]. The complexity of systems of care, individual practitioners, senior leadership [35], and strong organisational support and patronage have also been considered [36].

Since the study will be undertaken over a 2 year period, participants will be followed during the inpatient phase, through to the post-hospital and recovery phase of the injured child. This will enable the investigators to gain a better understanding of the changing needs and requirements of parents providing care and support for their injured children. The study aims to provide clear evidence of the unmet needs of parents and their children throughout the trauma journey. This information will also be used to inform the future development of a large scale, national, cross sectional study of the needs of families with children who have been severely injured.

## Dissemination

Study findings will be made available to participants, each study site, and the funders of this project, the Day of Difference Foundation. Research reports will be made available in peer-review publications, social media outlets, national and international conference presentations.

## Abbreviations

CD-RISC: Connor Davidson resilience scale
DASS 21: Depression, anxiety and stress scale
EQ-5D-Y: Quality of life standardised measure of health status
ISS: Injury severity score
PIP: Pediatric inventory for parents
SF 12v2: Parent quality of life
